# Automated gradient equilibration of macromolecular crystals to new solution conditions

**DOI:** 10.1107/S2053230X25008398

**Published:** 2025-10-03

**Authors:** Douglas H. Juers, Jack Quire, Sean Stothers

**Affiliations:** a Department of Physics Whitman College Walla Walla WA99362 USA; b Program in BBMB Whitman College Walla Walla WA99362 USA; MAX IV Laboratory, Sweden

**Keywords:** crystals, solutions, cracking, damage

## Abstract

We describe a device and a method for changing the ambient solution of a macromolecular crystal. The approach is gentle, automated, inexpensive and open source. Examples are given of the equilibration of three different crystals to new solutions with exchange times ranging from 5 to 180 min. In each case direct transfer of the crystal to the new solution causes cracking, which is eliminated with gradient equilibration using the described device. Crystals equilibrated with the device produce high-quality diffraction that yields refined structures comparable to those determined previously. The device offers a more systematic and labor-saving workflow than current practice both for performing diffraction analysis of macromolecular crystals and for investigating the response of macromolecular crystals to changes in solution composition.

## Abstract 2

A device and method for gentle, automated and inexpensive solution exchange for macromolecular crystals is described.

## 1. Introduction

Macromolecular crystals are commonly grown from a wide range of solution conditions, including aqueous solutions of salts, polyethylene glycols and alcohols. During growth, the solution becomes an integral part of the sample, permeating the crystal in nanometre-sized pores. Recognizing that the solution is important for maintaining crystalline order was a key development in the progression of X-ray diffraction analysis into a viable tool for structure determination of macromolecules (Bernal & Crowfoot, 1934 ▸).

Often after growth the crystal solution must be changed due to the overall experimental objectives or the requirements of downstream experimental details (Stum & Gleichmann, 1999 ▸). Some examples include changing the pH or salt conditions for functional reasons (López-Jaramillo *et al.*, 2002 ▸), adding a cryoprotective agent for low-temperature data collection (Garman & Doublié, 2003 ▸) and adding an inhibitor to the solution of an enzyme crystal (Hassell *et al.*, 2007 ▸; Müller, 2017 ▸; Kascakova *et al.*, 2025 ▸).

Changing the solution can damage the crystal in a variety of ways. The crystal may simply dissolve if the macromolecule is more soluble in the new solution, or the new condition may trigger an alternative packing arrangement that causes cracking (Perutz, 1953 ▸; Garman & Doublié, 2003 ▸; Kascakova *et al.*, 2025 ▸). Alternatively, the crystal may remain insoluble without obvious repacking in the new solution but suffer cracking from the rate of change of the solution conditions (López-Jaramillo *et al.*, 2002 ▸). Here, we will focus on reducing kinetic effects: that is, eliminating crystal damage that results from the rate of change of the crystal solution.

Several different techniques have been used to change crystal solutions. The simplest is to move the crystal directly into the new solution. In one early example, crystals were transferred from growth solutions containing high salt into organic solvents in a single step using a Pasteur pipet (Petsko, 1975 ▸). However, such direct single-step transfers are often unsuccessful, yielding cracked crystals that are unsuitable for diffraction analysis. In these cases, it often helps to reduce the concentration gradients experienced by the crystals. For example, direct transfer can still be used but in multiple steps using solutions of intermediate concentrations. In this case even very short soaks can be effective. Alternatively, the crystal can be left in place and the crystal solution can be changed via pipetting. This is less damaging than single-step direct transfer because the crystal experiences smaller concentration gradients (Garman & Owen, 2006 ▸), and repeated small additions can yield a very gentle overall gradient (Garman, 1999 ▸). Crystal manipulations can also be performed in a humid environment, which reduces the rate of evaporation from small droplets and limits crystal drying during transfer between drops (Farley *et al.*, 2014 ▸). Despite the effectiveness of these approaches, they involve a fair amount of handling with multiple pipettings and/or transfers, which create multiple opportunities to damage the crystals.

One way to reduce the required handling is with vapor diffusion, which has been employed to facilitate crystal transfer to new solutions. Crystals can be pre-equilibrated against the new solution via vapor diffusion, making subsequent liquid transfer more successful (Garman & Owen, 2006 ▸). Vapor diffusion can also directly deliver new solution components, as has been shown for quickly delivering volatile cryoprotective agents to loop-mounted crystals (Farley & Juers, 2014 ▸). Additionally, liquid droplets have been used to deliver ligands to crystals and change crystal solutions. This has been performed via acoustic droplet ejection (Collins *et al.*, 2017 ▸) and aerosolization (Ross *et al.*, 2021 ▸; Juers *et al.*, 2022 ▸).

Other researchers have shown that crystals grown in gels are more robust to soaking-induced damage (Sauter *et al.*, 2009 ▸; Sugiyama *et al.*, 2012 ▸). No doubt there are other inventive approaches that researchers have used over the years to successfully equilibrate their crystals to new solutions that have gone unreported.

Here, we focus on the traditional approach of gradually changing the crystal solution over time via repeated additions of a new solution to a small reservoir containing the crystals. Rather than manual pipetting, however, we employ an open-source syringe-pumping system capable of extremely low flow rates. The method is gentle, automated, inexpensive and open source.

## 2. Materials and methods

### 2.1. Chemicals and crystals

All proteins, crystallization reagents and other molecules were purchased from Sigma–Aldrich (St Louis, Missouri, USA). Crystals were grown using hanging-drop vapor diffusion in 24-well plates (Chryschem S Plates, Hampton Research) at 284–298 K and were used within a few weeks of growth. Reservoirs were 500 µl and drops were 6–18 µl. For tetragonal lysozyme (catalog No. L6876) the well contained 20 m*M* sodium acetate pH 4.5, 3–8%(*w*/*v*) NaCl and the protein solution was at 80 mg ml^−1^ in 20 m*M* sodium acetate pH 4.5 (Forsythe *et al.*, 1999 ▸). For thermolysin (catalog No. P1512) the well contained 2 *M* ammonium sulfate and the protein solution was at 100 mg ml^−1^ in 45%(*v*/*v*) DMSO (Hausrath & Matthews, 2002 ▸). For α-lactalbumin (catalog No. L5385) the well contained 50 m*M* KH_2_PO_4_, 15–20% PEG 8000 and the protein was at 30–50 mg ml^−1^ in water (Mueller-Dieckmann *et al.*, 2007 ▸). Crystal sizes were as follows. Lysozyme grew as chunks with edges of 100–600 µm. Thermolysin grew as rods of 300–1200 µm in length and 60–150 µm in diameter. α-Lactalbumin grew as thick plates of 100–200 µm in thickness and 500–1000 µm in the other dimensions.

### 2.2. Device fabrication

The auto gradient system is based on the poseidon open-source syringe-pump system (Booeshaghi *et al.*, 2019 ▸) and was fabricated using in-house facilities. Syringe-pump parts were printed in-house with FDM printers (Makerbot Replicator Gen 5, Raise3D Pro3, BambuLab A1 Mini) and PLA filament. The sample holder for the crystal pot and fluid tubes was designed using *OpenSCAD* and also 3D-printed in-house. The poseidon syringe-pumping software was modified with several options useful for crystal equilibration problems, including adding a menu to allow user-defined gradient flow, using *Qt Designer* (Qt Group, Espoo, Finland). The hardware and software are all open source, and detailed manufacture and assembly instructions are available on GitHub (Juers, 2024 ▸). The system employed here used the original poseidon syringe-pump design and the gradient flow software version 0.1.1 (poseidon_main_gf_0.1.1.py).

### 2.3. Crystal equilibration protocol

Once the crystal equilibration system has been built (Section 2.2), it can be used for crystal equilibration as follows.

#### 2.3.1. Syringe-pump preparation

To carry out the equilibration, the syringe-pumping system is first prepared with two syringes as follows.

(i) Both syringes (typically 1 or 3 ml; BD Biosciences, Franklin Lakes, New Jersey, USA) are fitted with a needle (21G from BD Biosciences or a blunt needle p/n 64-1490 from Warner Instruments, Holliston, Massachusetts, USA; Fig. 3).

(ii) Over the end of each needle, a small length (∼40 cm) of tubing (PE-50, Warner Instruments) is then carefully fitted.

(iii) Each syringe is then placed in a syringe-pump framework after setting the plunger transport at the appropriate position to accommodate the length of the plunger (Fig. 3).

(iv) The syringe pump is used to push solution into the tubing of the injection syringe until a small bead appears at the end of the tubing, which is blotted with a kimwipe.

(v) The syringe pump is used to pull a small volume (∼100 µl) of water into the tubing connected to the withdrawal syringe, followed by about 10 µl of air. This creates a buffer to prevent contamination when the withdrawal tubing is placed into the crystal pot. Note that for steps (iv) and (v) it is important to use the syringe pumps to move solution into and out of the tubing to ensure that when the gradient is executed the syringe plungers are already under compression or tension.

(vi) Navigate to the Auto Run menu and set up the desired gradient parameters. We often use gradients of 0–95% or 0–98% of the target solution.

#### 2.3.2. Crystal preparation

Next, the crystal(s) are prepared as follows.

**Figure 1:**
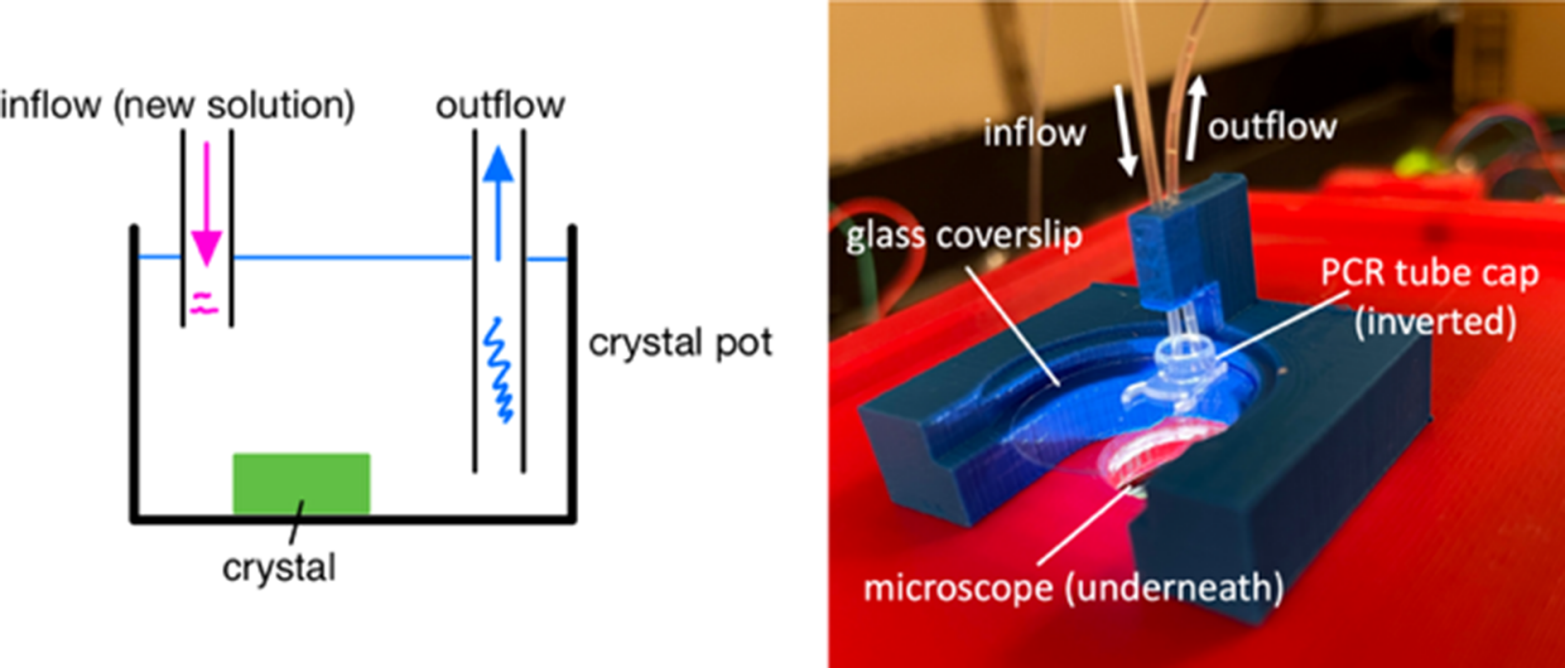
Left: schematic of equilibration setup. The crystal (green) sits in a 40 µl pot with two tubes used for the introduction of new solution and simultaneous withdrawal of the existing solution. Right: detail of the actual system. The crystal sits in the PCR tube cap, which sits on a glass cover slip. The blue housing holding the cover slip and tubing sits on a platform (red), which includes a port for the microscope.

(i) A crystal pot is prepared by using a razor blade to separate a single transparent PCR tube cap from a strip of caps (for example catalog No. 27-110, Genesee Scientific, Morrisville, North Carolina, USA) and inverting the cap. A small amount of starting solution is placed in the pot. We normally use 40 µl.

(ii) Using a cryoloop or a pipet, the crystal is transferred from the growth drop into the pot. Note should be taken of roughly where in the pot the crystals are sitting.

(iii) Using tweezers, the pot is placed on a cover slip (22 mm diameter from Hampton Research or 22 × 22 mm square from Avantor Sciences), which has been placed in the 3D-printed sample holder (Fig. 1 ▸).

(iv) The outflow tubing is threaded through the inner hole on the sample holder (Fig. 1 ▸) and then optionally through the inner hole on the pot cover (if using the pot cover; Supplementary Fig. S1). This tube is then positioned in the crystal pot near the bottom and edge of the pot in a place that does not contain crystals.

(v) The inflow tubing is threaded through the outer hole on the sample holder (Fig. 1 ▸) and then optionally through the outer hold on the pot cover (if using the pot cover; Supplementary Fig. S1). This tube is then positioned by slowly lowering the tube until is just touches the liquid surface, which should be apparent by a sudden shift of the meniscus. The end of the inflow tubing should be placed at or just below the surface, further from the crystals, rather than deep in the pot. This will allow the inflow solution to disperse before being encountered by the crystals, reducing the concentration gradients experienced by the crystals. If using the pot cover, it can be lifted above the pot while positioning the inflow tube to allow easy visualization.

#### 2.3.3. Gradient execution

Next, the camera is focused on the desired location in the reservoir (typically the crystal) and the gradient is executed. In the experiments described here we executed a gradient from 0 to 95% or 0 to 98% of the target solution. To ensure the crystal is exactly at the target solution the gradient can be executed again, or the crystal can be briefly transferred into a drop of the target solution. Here we performed the latter.

#### 2.3.4. Crystal analysis

Subsequently, the crystal can be mounted for diffraction analysis or stored for later use. The software includes options for a gradient that is linear over time (on average) and for a gradient in which equal volumes of solution are removed/added at each time point.

The solutions used for the experiments described here are shown in Table 1 ▸.

### 2.4. X-ray diffraction

Crystals to be analyzed by X-ray diffraction were mounted with 400/25 µm micromesh mounts (MiTeGen, Ithaca, New York, USA), blotted gently on a cover slip and mounted on the diffractometer goniometer at room temperature (294 K) either using microRT tubes (MiTeGen, Ithaca, New York, USA) with the target solution as the reference solution or under humid flow using a home-made humidity-control system at 7–8 l min^−1^ through a 9 mm diameter nozzle. For the latter mounting scheme, the relative humidities (RH) used were: lysozyme, 97% RH for all crystals; α-lactalbumin, 92% RH for the target solution, 98–99% for crystals mounted directly from the well. These humidities were chosen with a combination of estimating the molar fraction of water (Wheeler *et al.*, 2012 ▸) and previous experience of each crystal system.

Diffraction measurements were performed with an Oxford Diffraction Nova system, which includes a microfocus Cu *K*α X-ray source (49.3 kV, 0.8 mA) and an Onyx CCD detector. The crystal-to-detector distance was 65 mm.

For crystal-quality assessments, a strategy for a full data set was determined using *CrysAlis^Pro^* (Oxford Diffraction). The first and last 15 frames from the strategy were then collected, integrated and scaled with *CrysAlis^Pro^* to determine the mosaicity, unit cell and diffraction strength (measured with *I*/σ; see also supporting information).

Two data sets were measured. In set A, which included all three crystal types, crystals were mounted with microRT tubes and 0.3° oscillations were used. In set B, crystals were mounted in humid flow and 0.5° oscillations were used. Set B included orthorhombic α-lactalbumin and tetragonal lysozyme, but not hexagonal thermolysin because the humidities required for thermolysin (∼100%) were outside the range of our humidity-control device. Exposure times were 20 s for lysozyme and thermolysin and 30 s for α-lactalbumin, which diffracts more weakly than the other two crystals.

### 2.5. Structure determination

For structure determination, a strategy was determined with *CrysAlis^Pro^* and the detector was set so that the edge was about 0.3 Å resolution beyond the visible diffraction spots. The diffraction data were integrated with *CrysAlis^Pro^* and scaled using *AIMLESS* (Evans, 2006 ▸) to a resolution limit chosen based on CC_1/2_ ≥ 0.25 (Karplus & Diederichs, 2015 ▸). Structure determination was carried out with the *Phenix* package (Adams *et al.*, 2010 ▸). The lysozyme structure was determined using sulfur SAD with *Phaser* (McCoy *et al.*, 2007 ▸). For α-lactalbumin and thermolysin, molecular replacement was performed using *Phaser* (McCoy *et al.*, 2007 ▸) with starting models of PDB entries 1f6s (α-lactalbumin; Rudiño-Piñera *et al.*, 2002 ▸) and 8tln (thermolysin; Holland *et al.*, 1992 ▸). For α-lactalbumin a single chain was used, and *Phaser* was able to find six chains in the asymmetric unit. For thermolysin, the asymmetric unit is a single polypeptide chain. Following initial building or placement of the structure, multiple rounds of model inspection and adjustment (including building water molecules and ions) followed by refinement in *Phenix* were performed. For lysozyme, the anomalous signal was used to help place chloride ions.

## 3. Results and discussion

**Figure 2:**
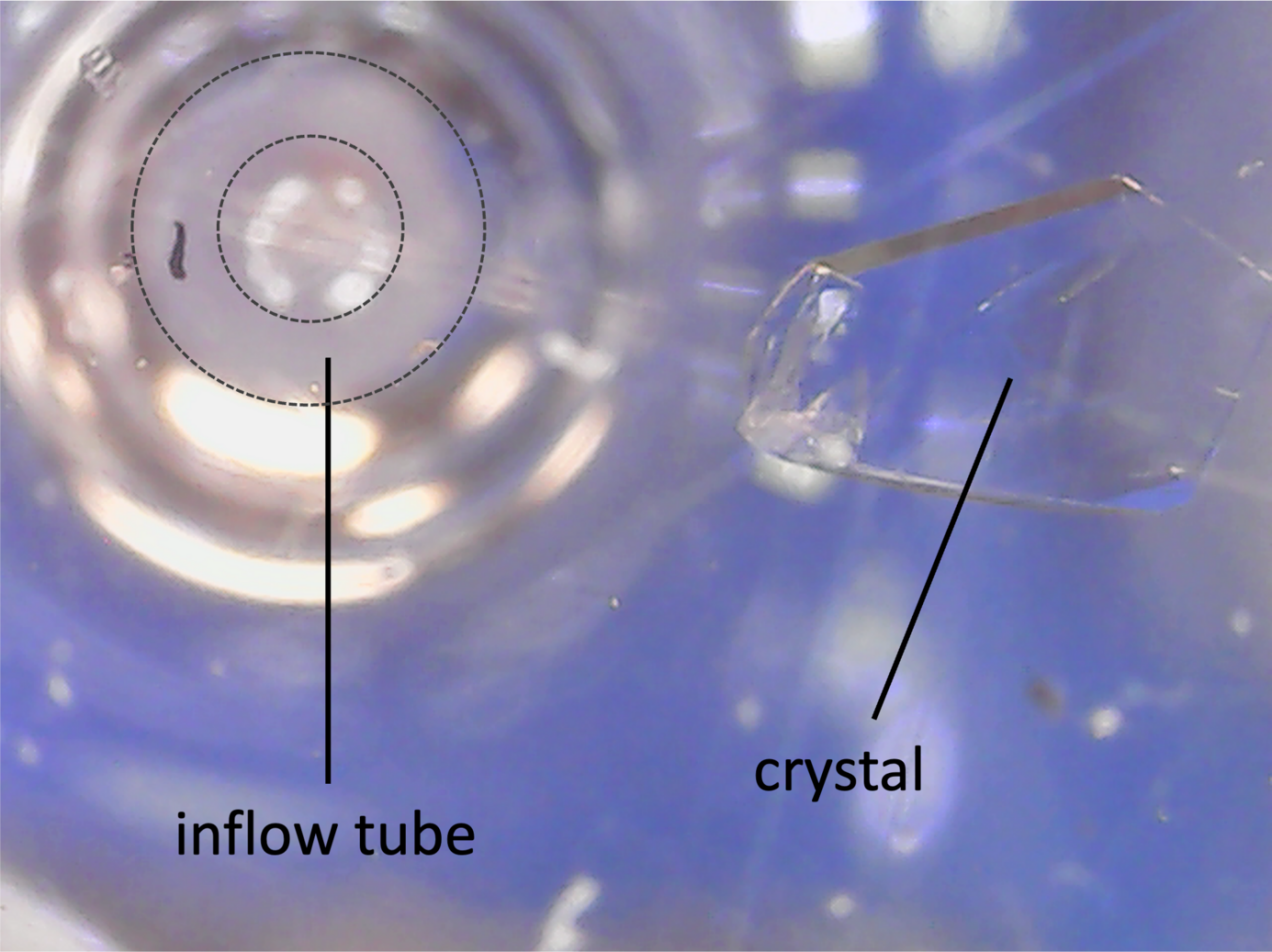
View of the pot through the microscope from below. The crystal sits on the right. The end of the circular inflow tube (white annulus) can be seen on the left.

Fig. 1 ▸ shows a schematic of the strategy and its physical realization in the current system. Fluid is simultaneously removed (outflow) and added (inflow) at user-defined rates, yielding gradual solution exchange. The arrangement of the inflow tube and the crystal can be adjusted depending on the relative density of the solutions. If, for example, the incoming solution has a greater density than the existing solution, we place the crystal offset to the side from the inflow tube so that the higher concentration solution does not simply fall directly on top of the crystal (Fig. 2 ▸). This measure is not always required, but can be helpful for particularly sensitive samples.

**Figure 3:**
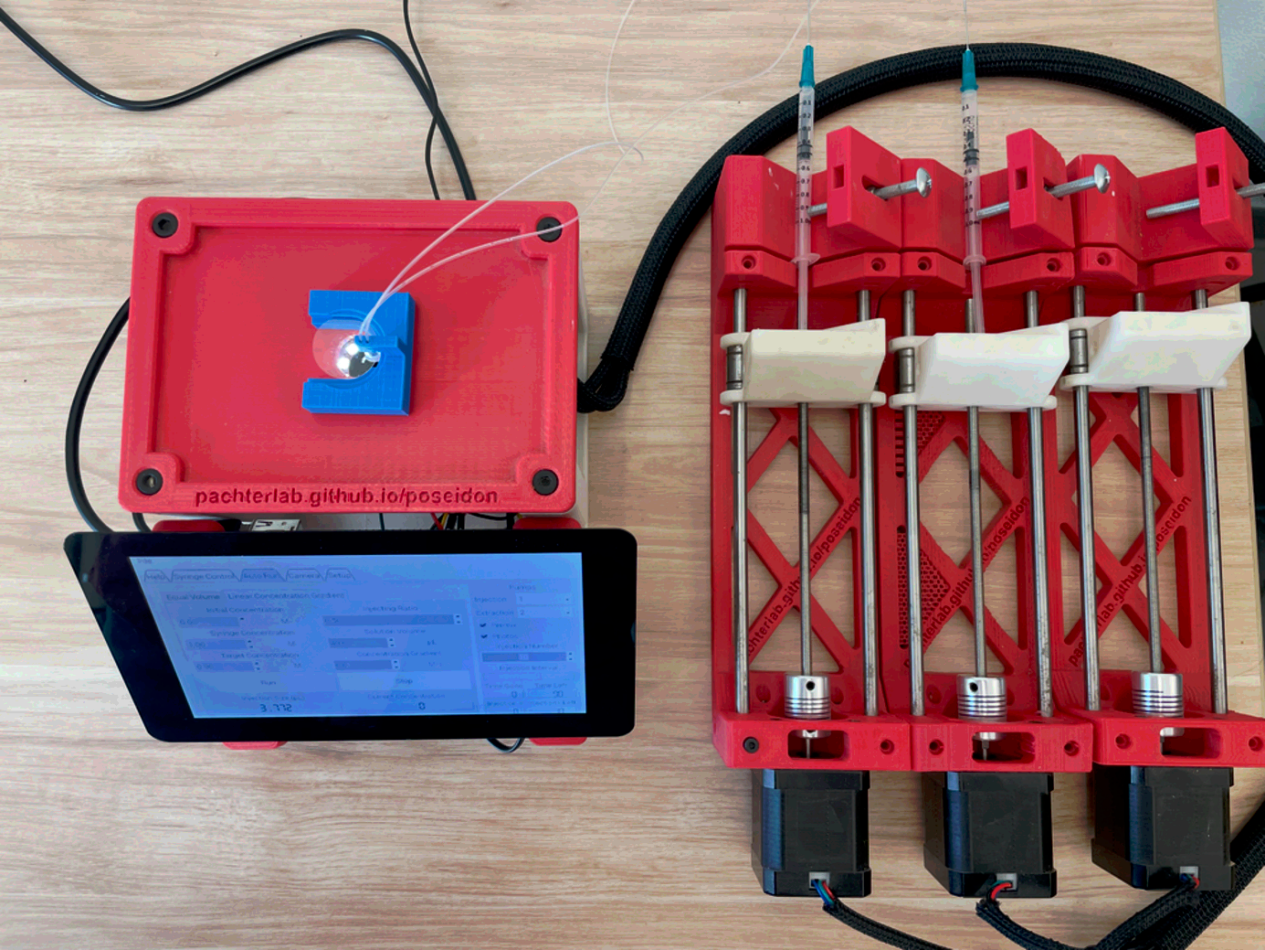
Overview of the complete system, showing the Raspberry Pi touch screen, red housing, the three syringe pumps and the blue housing which holds the PCR tube cap. The first two syringe pumps are loaded with syringes. Not visible in the image are the microscope, Arduino microcontroller with motor control shield, microscope (mounted below the red platform) and the Raspberry Pi (mounted on the back side of the touch screen). Each syringe pump is comprised of a black stepper motor coupled to a lead screw that moves the syringe plunger up and down via the white housing, which is threaded on the lead screw.

**Figure 4:**
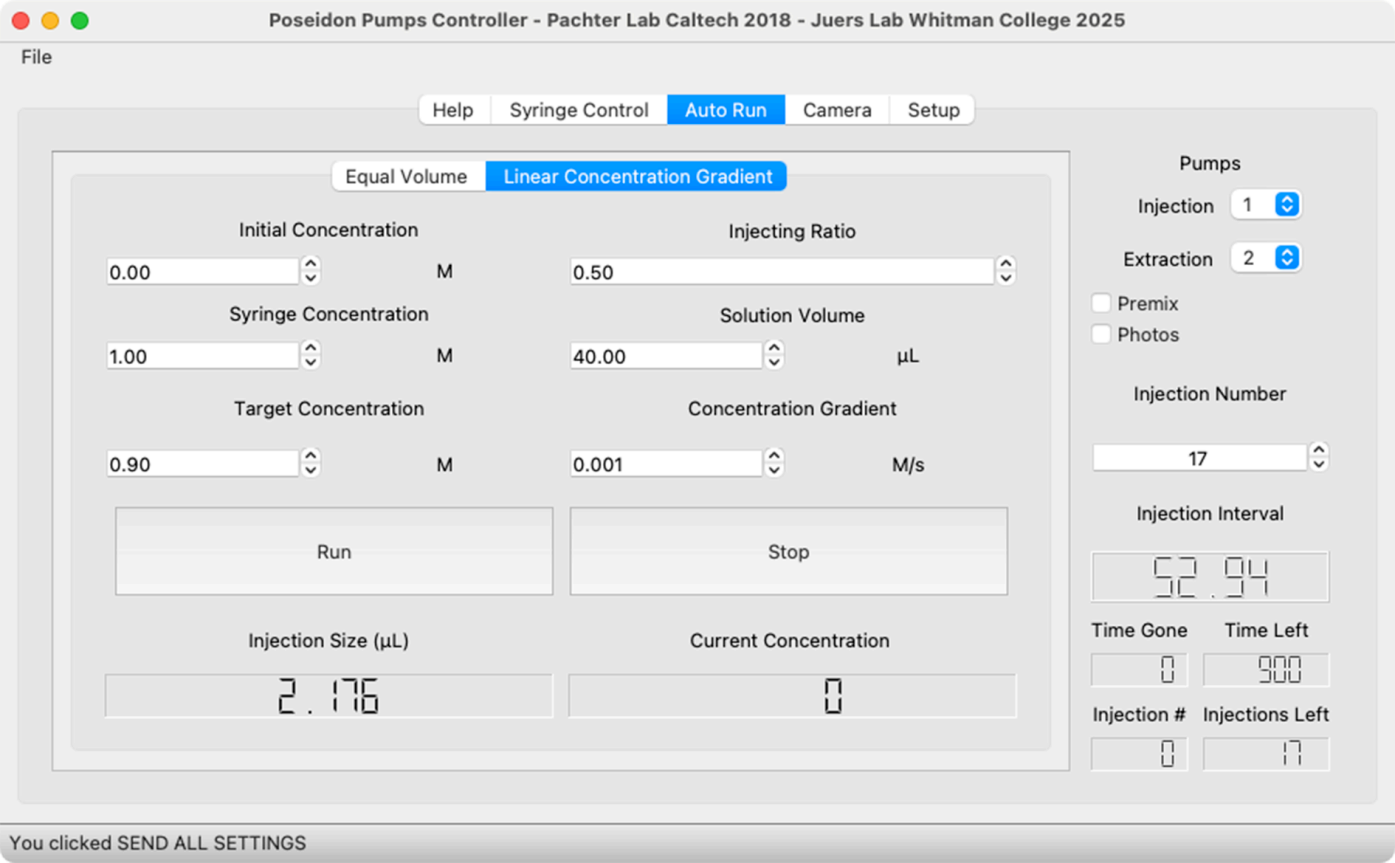
Software GUI showing the auto gradient menu.

Fluid flow is controlled with open-source syringe pumps (Fig. 3 ▸), which are constructed with stepper motors mounted in a framework. The rotation of the stepper motors is coupled to translation of a syringe plunger. Because stepper motors are very precise, the average flow rates of the syringe pumps can be very small. With a 1 ml syringe the smallest injection size is about 2 nl and flow rates of less than 100 nl h^−1^ are possible. The stepper motors are controlled with an Arduino microcontroller through an open-source Python program running on a desktop or laptop computer. Within the software, the user sets up a gradient flow system, setting the flow rates and rate of fluid exchange (Fig. 4 ▸). The system shown in Fig. 3 ▸ is controlled with a dedicated Raspberry Pi computer, which allows the system to be readily available when needed.

**Figure 5:**
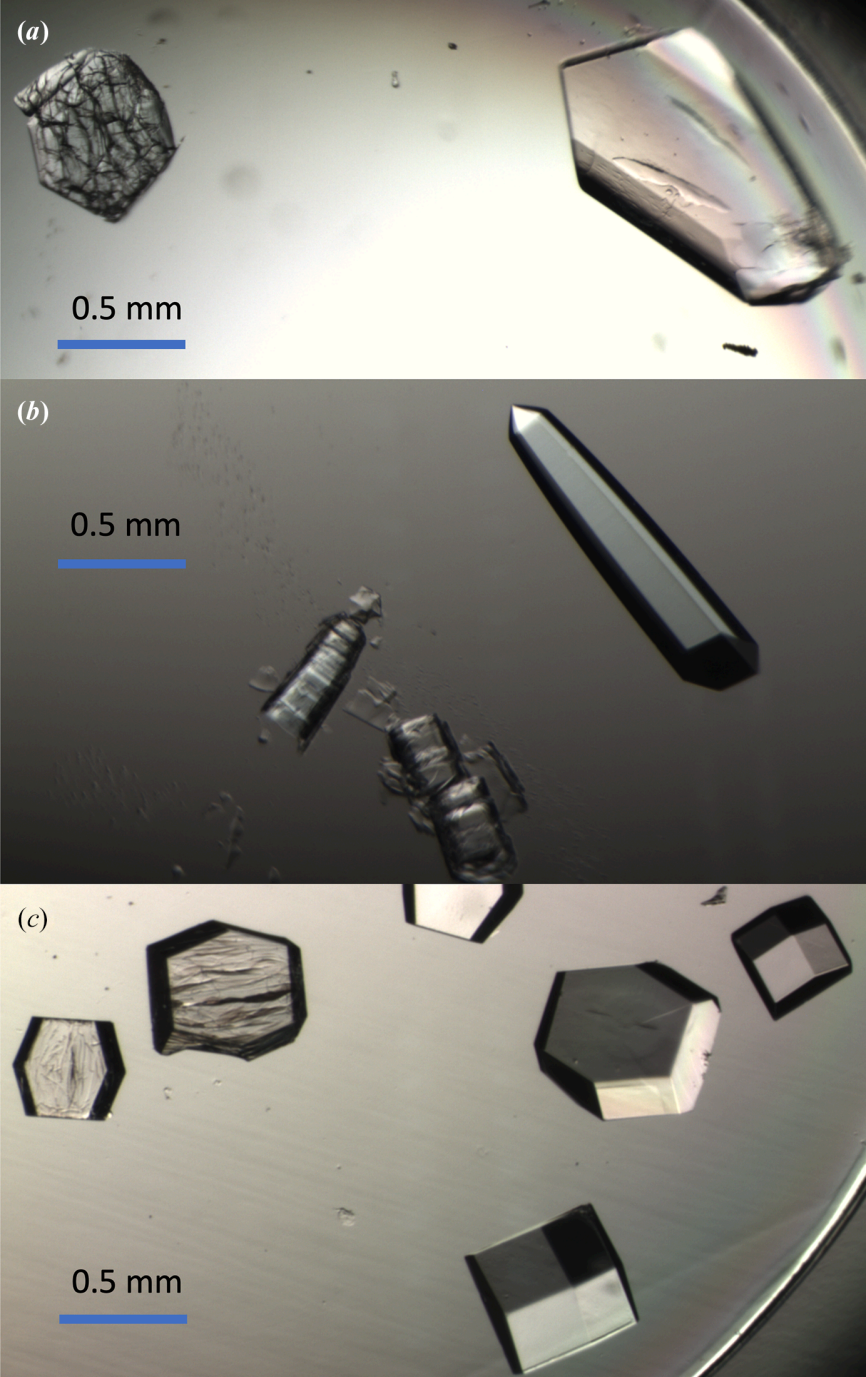
Examples of solution change using an automated linear gradient equilibration as described. Three different crystal pots are shown. In each panel, the intact crystals shown on the right were gradient-equilibrated and then transferred to a crystal pot containing the target solution. (*a*) α-Lactalbumin crystals, 0 → 25% glycerol over 50 min. (*b*) Thermolysin crystals, ∼2 *M* ammonium sulfate → water over 15 min. (*c*) Lysozyme crystals, 8% NaCl → 3% NaCl over 40 min. The cracked crystals shown on the left were directly transferred to the same pot with the target solution containing the gradient-equilibrated crystals. Hence, direct transfer to the target solution damages the crystals.

Fig. 5 ▸ shows some example crystal equilibrations, comparing single-step transfers with exchanges performed with the auto gradient system. The crystals sit in approximately 40 µl of solution, which is exchanged over time. In each case, direct transfer to the target solution yields cracked crystals. For each crystal type, a range of equilibration times was explored (Supplementary Table S1). We next summarize the salient results for each crystal system.

### 3.1. α-Lactalbumin

Fig. 5 ▸(*a*) shows crystals of an orthorhombic form of bovine α-lactalbumin in a glycerol-based cryosolution. The crystal on the right was gradually equilibrated from the well of 20% PEG 8K, 50 m*M* salt to the well + 25% glycerol. The crystal on the left was transferred directly to the glycerol solution, producing substantial cracking.

**Figure 6:**
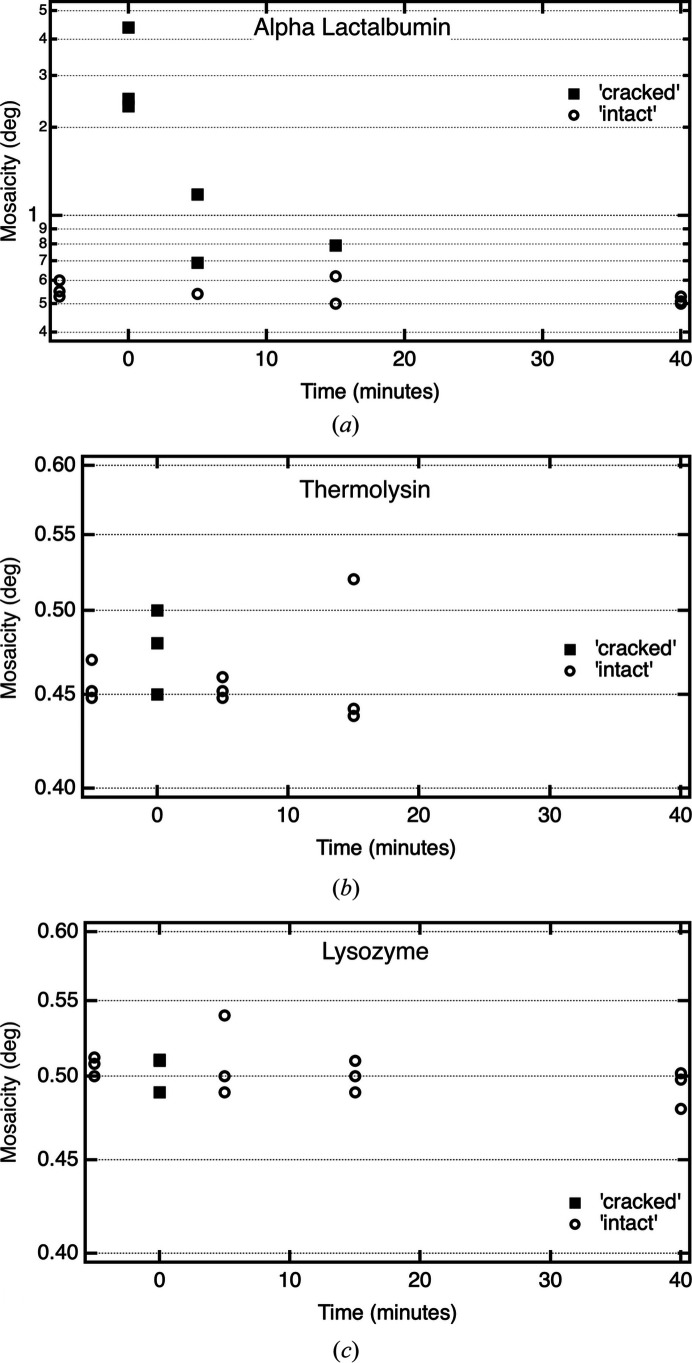
Dependence of mosaicity on gradient-equilibration time for the three crystals explored. Solid squares, crystals with visible cracks based on examination in a stereo microscope at ∼100×. Open circles, crystals that appear visually intact. (*a*) Orthorhombic α-lactalbumin, (*b*) hexagonal thermolysin, (*c*) tetragonal lysozyme,. The points plotted at *t* = −5 min are data for crystals mounted directly from the drop without treatment.

The cracked α-lactalbumin crystals diffracted weakly with very high mosaicity (Fig. 6 ▸, Supplementary Figs. S2 and S3), and sometimes autoindexing routines could not find a lattice with more than 50% agreement between the predicted and measured diffraction spots. Varying the gradient time shows that longer gradients reduce the probability of visually identifiable cracks, reduce the severity of cracking and reduce the mosaicity (Fig. 6 ▸*a*, Supplementary Fig. S2). The diffraction strength was also higher with longer equilibrations (Supplementary Fig. S3); however, the longer equilibrations also used larger crystals (Supplementary Fig. S4). Comparing shorter 15 min equilibrations that did not produce visible cracks with longer (40 or 45 min) equilibrations that also did not crack showed the same mosaicity (within uncertainties; Supplementary Table S2). Hence, whether more gentle gradients reduce mosaicity and/or increase diffraction power in visually intact crystals remains an open question.

We determined a room-temperature α-lactalbumin structure from a 200 × 400 × 900 µm crystal that was gradient-equilibrated to the glycerol solution over 40 min (Table 2 ▸). The structure was determined via molecular replacement to 2.2 Å resolution with a final refined *R*_work_ and *R*_free_ of 0.20 and 0.24, respectively. These indicators are similar to a room-temperature structure already deposited in the PDB, which was the starting model for molecular replacement (PDB entry 1f6s, 2.2 Å resolution, *R*_work_/*R*_free_ = 0.22/0.25). The α-carbons from the six chains in the refined model overlay with the six chains in PDB entry 1f6s with r.m.s.d. values of 0.4–0.9 Å.

### 3.2. Thermolysin

Fig. 5 ▸(*b*) shows equilibration of the hexagonal crystal form of thermolysin to pure water. Thermolysin has remarkably low solubility at low salt to the point that crystals are stable in small volumes of deionized water. Here, the crystals started in 2 *M* ammonium sulfate, which is similar to the well solution, and were equilibrated to deionized water with a linear gradient over 15 min. Single-step transfer breaks the crystal into small slabs. Individual slabs, although very small (∼70 × 20 µm), still diffracted to about 3 Å resolution with mosaicities similar to untreated crystals (Fig. 6 ▸*b*). A 5 min gradient equilibration to water is enough to prevent the breakup into slabs, leaving the crystal intact with a similar mosaicity and diffraction strength to untreated crystals. As with α-lactalbumin, the average mosaicities for shorter (5 min) and longer (15 min) equilibrations are the same (within uncertainties; Supplementary Table S2).

We determined a room-temperature thermolysin structure from a hexagonal rod (500 µm long with a diameter of 75 µm) that was gradient-equilibrated over 15 min to 100 m*M* ammonium sulfate (Table 2 ▸). The structure was determined via molecular replacement to 1.75 Å resolution with a final refined *R*_work_ and *R*_free_ of 0.17 and 0.19, respectively. These values are similar to those of other room-temperature thermolysin structures in the PDB (49 RT structures with 〈*R*_work_〉 = 0.16, 〈*R*_free_〉 = 0.20 and 〈resolution〉 = 1.96 Å). The refined model α-carbons overlay with the starting model (PDB entry 8tln) with an r.m.s.d. value of 0.13 Å.

### 3.3. Lysozyme

Fig. 5 ▸(*c*) shows the equilibration of crystals of the tetragonal crystal form of hen egg-white lysozyme to lower salt conditions. Lysozyme is a model system for investigations of the response of crystals to changes in solution conditions (Lopez-Jaramillo *et al.*, 2002 ▸). Here, the starting solution was the well solution consisting of 40 m*M* sodium acetate pH 4.5, 8% NaCl and the final solution was 40 m*M* sodium acetate, 3% NaCl. Direct transfer produces roughly parallel cracks due to solvent movement into the crystal under hypotonic conditions (López-Jaramillo *et al.*, 2002 ▸), while a gradient equilibration of 5 min eliminated the cracking. Lysozyme crystals sometimes showed surface degradation during the equilibrations, so the target solution was supplemented with 5 mg ml^−1^ protein. Diffraction experiments showed there was not a clear dependence of either the mosaicity or the diffraction strength on the gradient time (Fig. 6 ▸*c*, Supplementary Figs. S2 and S3 and Table S2). Quite surprisingly, the crystals directly transferred to 3% NaCl (which cracked similarly to Fig. 5 ▸) diffract with a similar mosaicity and diffraction strength to both gradient-equilibrated crystals and the original untreated crystals (Fig. 6 ▸*c*, Supplementary Figs. S2 and S3). Even a crystal subjected to a more aggressive treatment (direct transfer to 0.75% NaCl), which cracked more extensively, showed a mosaicity (0.50°) and diffraction strength (〈*I*/σ〉 = 13.8 at 2.10–2.00 Å) on a par with the other crystals.

We determined a room-temperature lysozyme structure from a 100 × 300 × 600 µm crystal that was gradient-equilibrated to 3% NaCl over 40 min (Table 2 ▸). The structure was determined via sulfur SAD to 1.30 Å resolution with a final refined *R*_work_ and *R*_free_ of 0.16 and 0.18, respectively. The refined model α-carbons overlay with another 1.3 Å resolution room-temperature structure (PDB entry 193l) with an r.m.s.d. value of 0.15 Å.

In each case described, the crystals can be equilibrated to the new solution via direct transfer in smaller concentration steps or by repeated manual pipetting. However, the repeated transfers require constant attention, and present more opportunity for something to go wrong and for the crystals to be damaged. Here, there is only one transfer needed to equilibrate to the new solution, and once the gradient is started the crystal(s) can be left unattended while the user’s attention can be directed elsewhere as the crystals are equilibrating. The solution gradient may be as shallow as required to yield an intact crystal.

### 3.4. Equilibration time and evaporation

Here, we found that equilibration times of <60 min were adequate. For the lysozyme and thermolysin crystals, just a 5 min gradient was sufficient to prevent visible cracking, while α-lactalbumin benefitted from more gradual treatments. For particularly sensitive samples, including larger crystals, one could imagine longer gradients being employed. At the humidity of our laboratory (30–40% RH), the evaporation rate of water from the open pot of Fig. 1 ▸ is about 6 µl h^−1^. We fashioned a cover to go over the pot (with ports for the tubing), which reduces this rate to about 1 µl h^−1^ (Supplementary Fig. S1). Using this cover, we performed 3 h gradient equilibrations of α-lactalbumin to the 25% glycerol solution. These crystals showed some surface degradation, but yielded mosaicities similar to the shorter equilibrations (0.54–0.57°). Thus, there is potential for orders-of-magnitude slower equilibrations than those used here.

It is certainly possible that crystal order and diffraction quality could be compromised even though visually the crystal seems intact. Our results are inconclusive on this question, requiring more extensive analysis of more samples and possibly employing a less divergent beam, since for many of our samples the mosaicity is probably dominated by instrument broadening rather than crystal characteristics. However, this system creates the opportunity for more systematic investigations of the effects of equilibration rate on crystal order.

Here, we used a reservoir volume of 40 µl. Although small, this volume is still large enough that in some cases surface degradation is caused by dissolution of the crystals when placed in the reservoir. This can be prevented by adding a low concentration of the protein to the initial and/or final solutions, as we did for lysozyme here.

In conclusion, we have assembled a system to facilitate crystal solution exchange with minimal handling. X-ray diffraction from crystals equilibrated with the system yields high-quality refined structures. Inexpensive and straight­forward to build using open-source components, the system should be a useful tool in the workflow of structural biology laboratories. Additionally, the tool may facilitate more systematic studies of the responses of crystals to solution concentration gradients.

## 4. Related literature

The following references are cited in the supporting information for this article: Kabsch (2001 ▸, 2010 ▸).

## Supplementary Material

PDB reference: α-lactalbumin, 9ozw

PDB reference: thermolysin, 9ozy

PDB reference: lysozyme, 9oz5

Supplementary Methods, Tables and Figures. DOI: 10.1107/S2053230X25008398/yg5013sup1.pdf

## Figures and Tables

**Table 1:** Solutions used for equilibrations

Crystal	Starting solution (well)	Target solution
α-Lactalbumin	12–20% PEG 8K, 0–125 m*M* KH_2_PO_4_	20% PEG 8K, 50 m*M* KH_2_PO_4_, 25% glycerol
Lysozyme†	7–9% NaCl, 20 m*M* sodium acetate pH 5.2 or 4.5	3% NaCl, 20 m*M* pH 5.2 or 4.5, 5 mg ml^−1^ lysozyme
Thermolysin	1.7–2.1 *M* ammonium sulfate	Water or 100 m*M* ammonium sulfate‡

† The crystals shown in Fig. 5 ▸ used pH 4.5 and had no lysozyme in the target solution. The crystals used for diffraction analysis used pH 5.2 and included lysozyme in the target solution.

‡ Water for crystal quality assessments and ammonium sulfate for structure determination.

**Table 2:** Diffraction and refinement statistics

Protein	α-Lactalbumin	Thermolysin	Lysozyme
Crystal dimensions (µm)	900 × 400 × 200	75 in diameter, 500 in length	400 × 400 × 200
Equilibration solution	20% PEG 8K, 50 m*M* KH_2_PO_4_, 25% glycerol	100 m*M* ammonium sulfate	3% NaCl, 20 m*M* sodium acetate pH 5.2
Equilibration time (min)	40	15	40
Wavelength (Å)	1.54	1.54	1.54
Oscillation range (°)	0.5	0.3	0.3
Total range (°)	75	78	292
Exposure time (s)	30	60	60
Resolution range (Å)	29.59–2.17 (2.24–2.17)	29.71–1.75 (1.78–1.75)	28–1.30 (1.32–1.30)
Space group	*P*2_1_2_1_2	*P*6_1_22	*P*4_3_2_1_2
*a*, *b*, *c* (Å)	72.296, 105.084, 118.19	93.572, 93.572, 131.042	79.182, 79.182, 37.985
α, β, γ (°)	90, 90, 90	90, 90, 120	90, 90, 90
Total reflections	141407 (10864)	177295 (5744)	268417 (9472)
Unique reflections	47425 (4154)	34729 (1851)	38312 (1460)
Multiplicity	3.0 (2.6)	5.1 (3.1)	8.9 (6.5)
Completeness (%)	98.0 (99.9)	99.7 (99.8)	100.00 (100.00)
Mean *I*/σ(*I*)	7.8 (0.9)	6.1 (0.7)	20.9 (1.2)
Wilson *B* factor (Å^2^)	41.3	19.5	16.3
*R*_merge_	0.071 (0.988)	0.186 (1.671)	0.049 (2.816)
*R*_meas_	0.087 (1.233)	0.205 (2.017)	0.052 (3.053)
*R*_p.i.m._	0.049 (0.721)	0.083 (1.103)	0.017 (1.161)
CC_1/2_	0.999 (0.407)	0.982 (0.341)	1.000 (0.354)
Reflections used in refinement	47364 (2774)	34648 (2584)	30268 (2691)
Reflections used for *R*_free_	2378 (148)	1787 (131)	1525 (140)
*R*_work_	0.1966 (0.3264)	0.1586 (0.3412)	0.1636 (0.3328)
*R*_free_	0.2375 (0.3745)	0.1871 (0.3518)	0.1755 (0.3514)
No. of non-H atoms			
Total	5904	2706	1145
Macromolecules	5860	2455	1044
Ligands	6	9	2
Solvent	38	242	99
Protein residues	732	318	129
R.m.s.d., bond lengths (Å)	0.007	0.007	0.005
R.m.s.d., angles (°)	0.93	0.91	0.84
Ramachandran favored (%)	94.17	96.82	99.21
Ramachandran allowed (%)	5.56	3.18	0.79
Ramachandran outliers (%)	0.28	0	0
Rotamer outliers (%)	4.35	0.39	1.79
Clashscore	3.5	2.31	0.97
Average *B* factor (Å^2^)			
Overall	50.01	22.46	22.72
Macromolecules	50.1	21.2	21.7
Ligands	38.3	34.21	24.13
Solvent	37.48	34.81	33.55
PDB code	9ozw	9ozy	9oz5
