# Supplementary material for: Automated gradient equilibration of macromolecular crystals to new solution conditions
Source: Acta Crystallogr F Struct Biol Commun. 2025 Oct 3;81(Pt 11):478–86. doi: 10.1107/S2053230X25008398 (PMC12576687; doi:10.1107/S2053230X25008398)
Supplement: Supplementary file 1 [file f-81-00478-sup1.pdf]

STRUCTURAL BIOLOGY
COMMUNICATIONS

Volume 81 (2025)

Supporting information for article:

Automated gradient equilibration of macromolecular crystals to new solution conditions

Douglas H. Juers, Jack Quire and Sean Stothers

Automated Gradient Equilibration of Macromolecular Crystals to New Solution Conditions

Juers, Quire & Stothers.

Supplementary Information

Supplementary methods - Mosaicity

In CrysAlisPro the 'mosaicity' is given as three components, e1, e2 and e3, which are the mosaicities in three directions defined in a coordinate system local to eachreflection. e1 and e2 are the mosaicities (i.e. the angle subtended by the diffraction spots) in two orthogonal directions tangential to the Ewald sphere (on the image, e2 is themosaicity along the direction radial from the beam centre), while e3 is the mosaicity in a direction perpendicular to e1 and s - s0, which is roughly the mosaicity in the scanning direction, where s and s0 are the scattered and incident X-ray vectors, respectively [1]. The e3 mosaicity parameter is similar to the REFLECTING_RANGE parameter in XDS. For the crystals tested here, the e3 values are about six times greater than the REFLECTING_RANGE_E.S.D., which is the value reported by XDS as the mosaicity [2]. Here we use the e3 value as the mosaicity.

Supplementary Tables

Table S1. Crystal condition from visual inspection after equilibration. Crystals were visualized with a stereomicroscope after gradient equilibration prior to mounting for X-ray analysis, and judged whether cracks were present or absent. The samples are broken into two groups. Set A = 0.3° oscillations/microRT tube; set B = 0.5 ° oscillations/RH flow.

Crystal	Equil Time	Cracked/Total (set A)	Cracked/Total (set B)	Cracked/Total (summed)
Alpha lactalbumin	0	3/3	3/3	6/6
	5	2/3	3/3	5/6
	15	1/3	1/3	2/6
	40	0/3	-	0/3
	45	-	0/3	0/3
	None	0/3	0/3	0/6
Lysozyme	0	3/3	4/4	7/7
	5	0/3	0/3	0/6
	15	0/3	1/5	1/8
	40	0/3	0/5	0/8
Thermolysin	0	3/3		
	5	0/3		
	15	0/3		
	None	0/3		

Table S2. Comparing mosaicities of visually intact crystals to explore the idea that gentler equilibrations yield higher quality diffraction even in the absence of visually apparent crystal damage. SE = standard error.

Crystal	Equil Time	Avg Mosaicity (SE)
Alpha lactalbumin	15 (set A)	0.56 (6)
	40 (set A)	0.52 (1)
	15 (set B)	0.62 (2)
	40 (set B)	0.64 (2)
Lysozyme	5 (set A)	0.510 (15)
	15 (set A)	0.500 (6)
	40 (set A)	0.493 (7)
	5 (set B)	0.650 (26)
	15 (set B)	0.620 (10)
	40 (set B)	0.638 (20)
Thermolysin	5 (set A)	0.453 (3)
	15 (set A)	0.508 (45)

Supplementary Figures

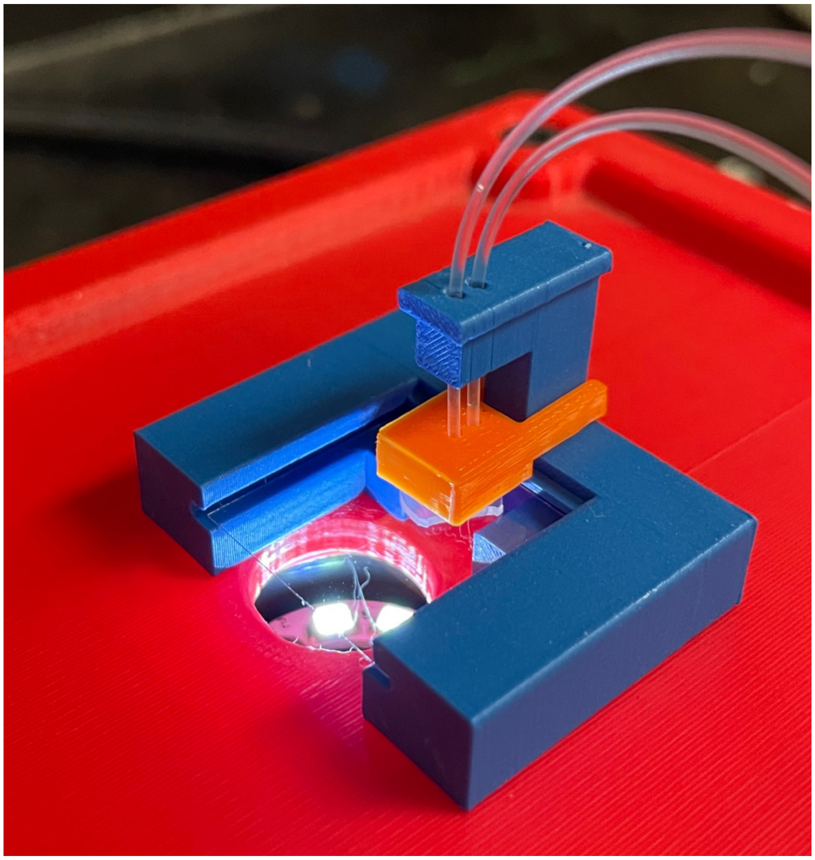

Fig S1. Sample holder with cover. The sample holder shown uses square coverslips (22 mm on a side), and can accommodate a cover for the crystal pot. Use of the cover reduces the evaporation rate of water from the pot by about 80%.

Crystal characteristics plots. Crystal characteristics (mosaicity, I/sigma, crystal volume, cell edge lengths) are plotted against the equilibration time. Each plotted point is an average of 3-5

crystals, and the error bars are standard error. Upper: Set A(0.3° oscillations with crystals mounted with microRT tubes. Lower: Set B (0.5° oscillations with crystals mounted in humid flow). The points plotted at t = -5 minutes are data for crystals mounted directly from the drop without treatment. In each plot, alact=orthorhombic alpha-lactalbumin, lyz=tetragonal lysozyme, tln=hexagonal thermolysin.

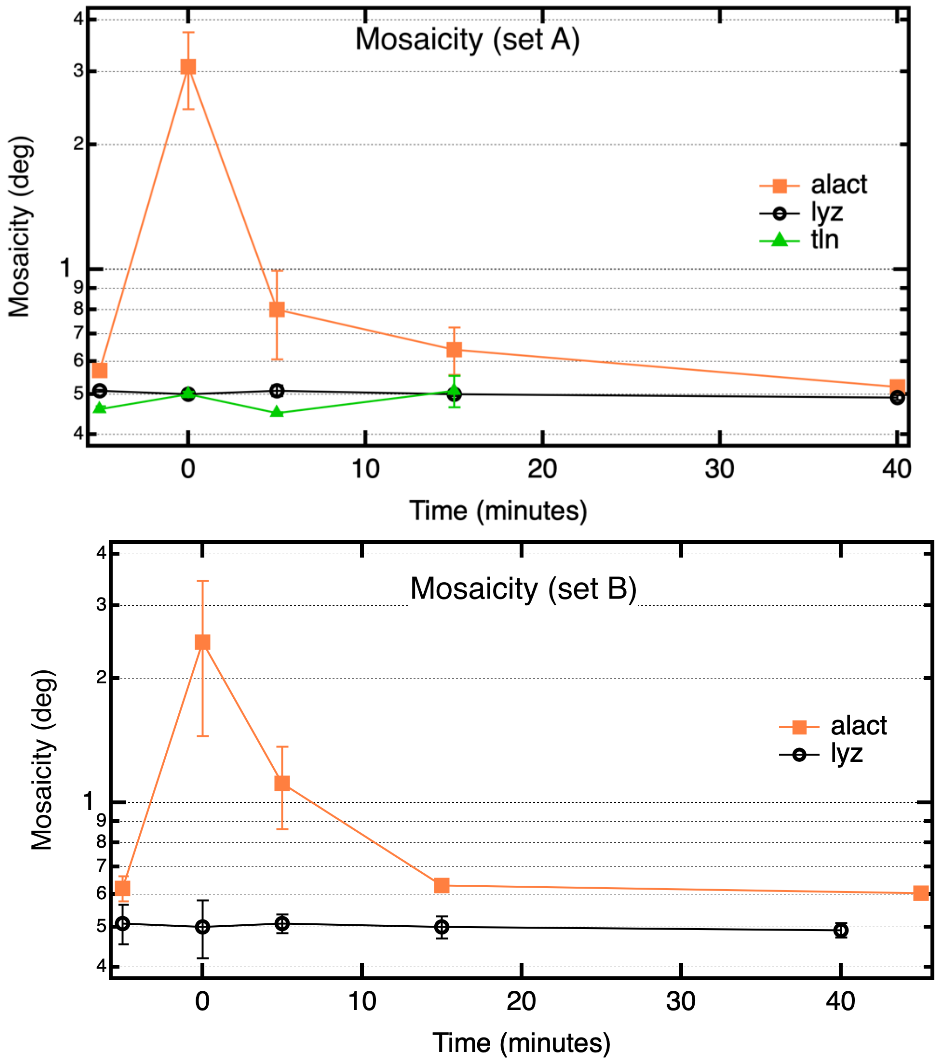

Figure S2. Mosaicity vs equilibration time.

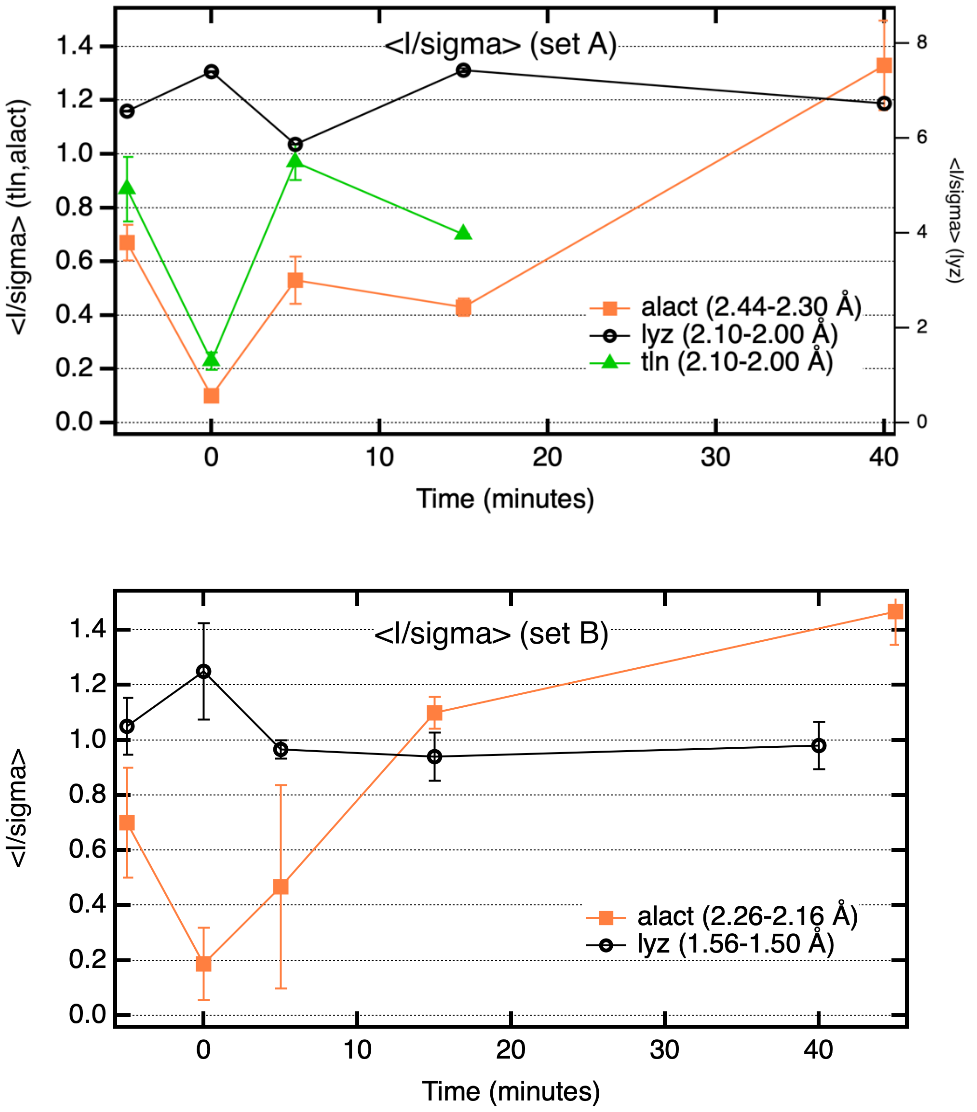

Figure S3. <I/sigma> in a specific resolution shell (stated in the legend) vs equilibration time.

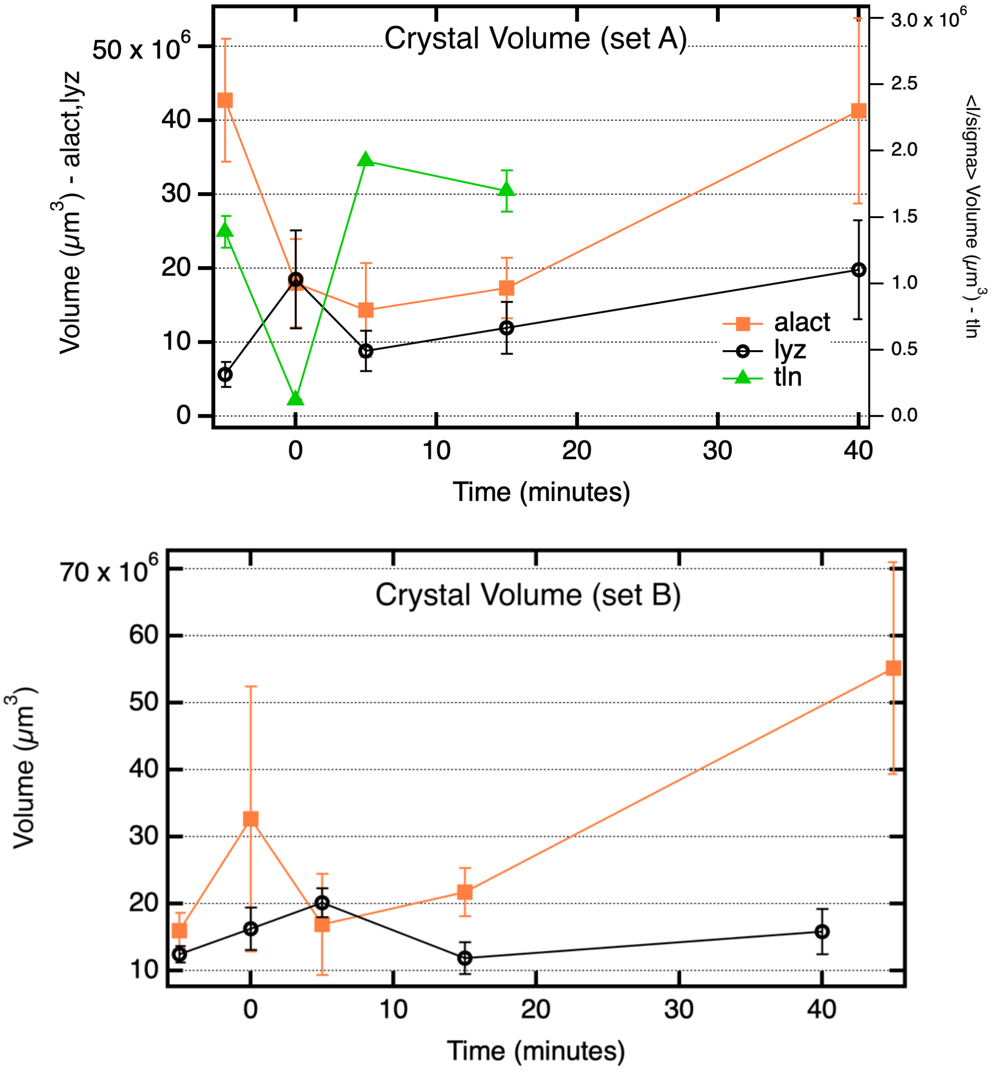

Fig S4. Estimated crystal volume, based on crystal dimensions discerned from the alignment microscope on the diffractometer vs equilibration time.

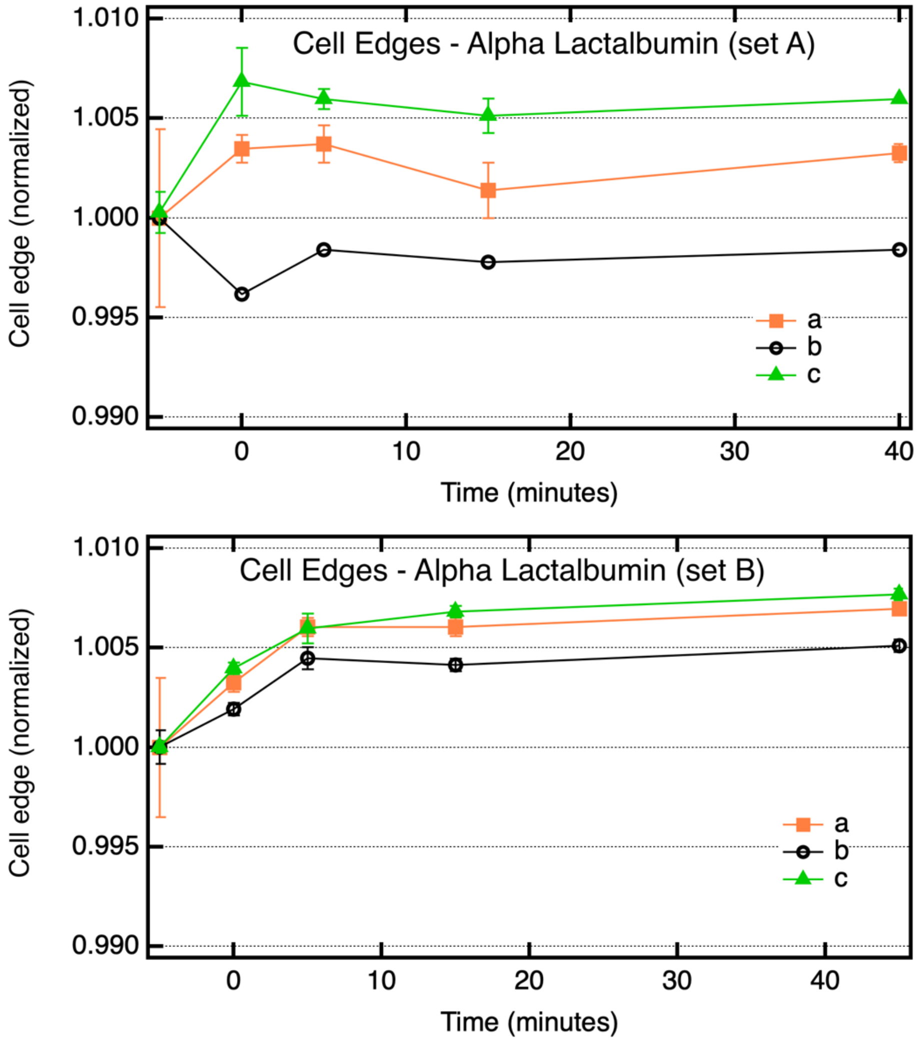

Fig S5a. Alpha lactalbumin cell edges, normalized to the averaged untreated crystal values, which are 71.9 (3) Å, 104.7 (1) Å and 117.2 (1) Å for set A and 71.8 (3) Å, 104.5 (1) Å and 117.27 (3) Å for set B.

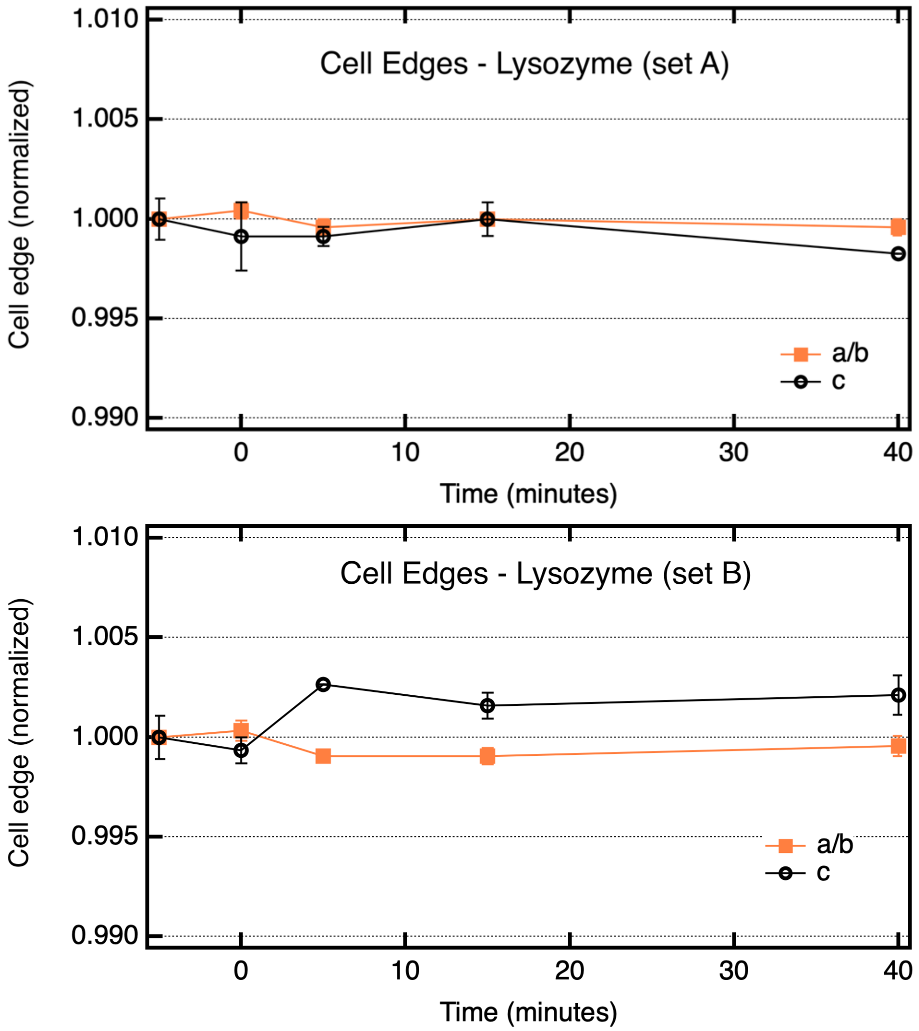

Fig S5b. Normalized Tetragonal lysozyme crystal cell edges. Values for untreated crystals are a=b=79.2Å, c=38.0 Å for both sets A and B.

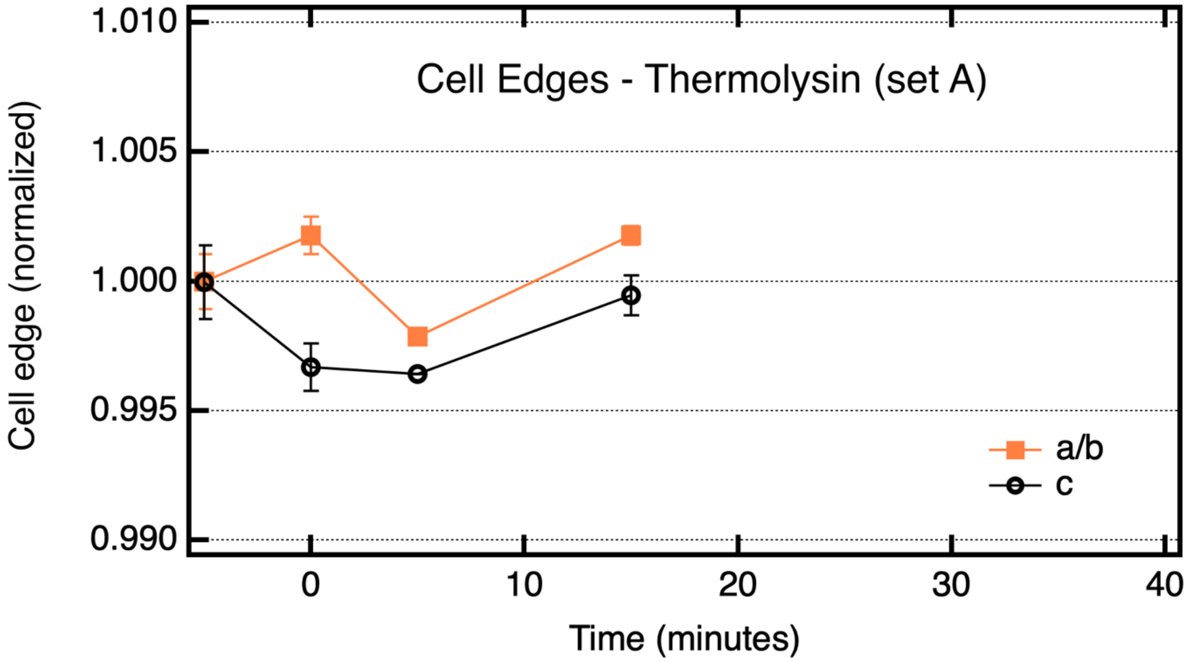

Fiig S5c. Normalized hexagonal thermolysin crystal cell edges. Values for untreated crystals are a=b=93.6 (1) Å, c=131.3 (2) for set A.

[1] Kabsch W. International Tables of Crystallography, Vol F2001. p. 218-25.
[2] Kabsch W. Integration, scaling, space-group assignment and post-refinement. Acta Crystallogr D Biol Crystallogr. 2010;66:133-44.
